# The Manifold Roles of Sphingolipids in Viral Infections

**DOI:** 10.3389/fphys.2021.715527

**Published:** 2021-09-29

**Authors:** Elita Avota, Jochen Bodem, Janice Chithelen, Putri Mandasari, Niklas Beyersdorf, Jürgen Schneider-Schaulies

**Affiliations:** Institute for Virology and Immunobiology, University of Würzburg, Würzburg, Germany

**Keywords:** sphingolipid, ceramide, sphingosine-1-phosphate, plasma membrane, virus entry, virus replication, virus budding

## Abstract

Sphingolipids are essential components of eukaryotic cells. In this review, we want to exemplarily illustrate what is known about the interactions of sphingolipids with various viruses at different steps of their replication cycles. This includes structural interactions during entry at the plasma membrane or endosomal membranes, early interactions leading to sphingolipid-mediated signal transduction, interactions with internal membranes and lipids during replication, and interactions during virus assembly and budding. Targeted interventions in sphingolipid metabolism – as far as they can be tolerated by cells and organisms – may open novel possibilities to support antiviral therapies. Human immunodeficiency virus type 1 (HIV-1) infections have intensively been studied, but for other viral infections, such as influenza A virus (IAV), measles virus (MV), hepatitis C virus (HCV), dengue virus, Ebola virus, and severe acute respiratory syndrome coronavirus type 2 (SARS-CoV-2), investigations are still in their beginnings. As many inhibitors of sphingolipid metabolism are already in clinical use against other diseases, repurposing studies for applications in some viral infections appear to be a promising approach.

## Introduction

**Figure 1:**
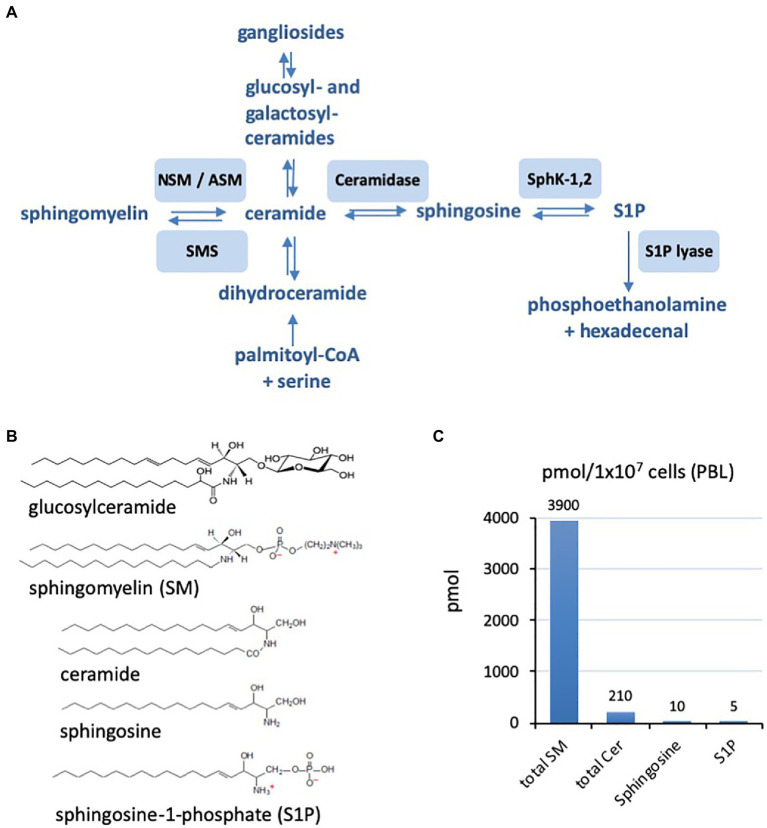
**(A)** Schematic representation of the sphingolipid metabolism from *de novo* synthesis from palmitoyl-CoA and serine to the final degradation to phosphoethanolamine and hexadecenal. Involved enzymes are neutral and acid sphingomyelinase (ASM; neutral sphingomyelinase, NSM), sphingomyelin synthetase (SMS), ceramidase, sphingosine kinases 1 and 2 (SphK-1,2), and sphingosine-1-phosphate (S1P) lyase. **(B)** Examples of the chemical structures of sphingolipids with sphingomyelin (SM) and ceramides of chain length 16 (C16). **(C)** Characteristic ratios of amounts of SM, ceramide (Cer), sphingosine, and sphingosine-1-phosphate (S1) in primary human peripheral blood lymphocytes (PBL; example form our own research).

As obligate intracellular pathogens, viruses must interact with and overcome cellular membranes as a key step of their life cycle. Sphingolipids (Figure 1) are a major structural component of the cellular plasma membrane but also act as bioactive lipids transducing signals intracellularly as well as to other cells. Their roles in uninfected and infected host cells, with and without specific inhibitors of the sphingolipid metabolism, have been studied increasingly using advanced quantitative analysis methods and microscopical localization (for recent reviews also see: Schneider-Schaulies and Schneider-Schaulies, 2015; Muller et al., 2019; Beckmann and Becker, 2021). Targeted manipulations of the host cell sphingolipid metabolism might be exploited to limit viral replication and thus open novel therapeutic options. This review will focus on examples of how the viral replication cycle can be affected by targeting sphingolipid metabolism. Sphingolipids influence the following three steps of viral replication: (1) structural consequences at membranes influencing, for example, the fusion of the plasma membrane with the viral membrane, (2) consequences for cellular processes like endocytosis also supporting the endocytosis of viruses, and (3) signaling and consequences for the cellular metabolism influencing the viral replication cycle. As each individual virus exploits a certain set of properties of its target cells, the roles of sphingolipids are different and must be investigated in detail for each virus.

## Sphingolipid Interactions During Viral Attachment and Entry

The first steps of the viral life cycle are attachment and entry, which enable the pathogen’s passage through the host cell plasma membrane and subsequent uncoating and release of viral nucleocapsids into the cytoplasm. These steps are predominantly influenced by membrane microdomains enriched for particular sphingolipid species, which act *via* segregating receptors and modulating biophysical processes such as membrane fusion or endocytosis and subsequent fusion. In addition, these first interactions between viruses and cells may induce signaling cascades which affect uptake, intracellular trafficking and replication of viruses (for a schematic representation of involved pathways, see Figure 1).

### Glycosphingolipids in Viral Entry

**Figure 2:**
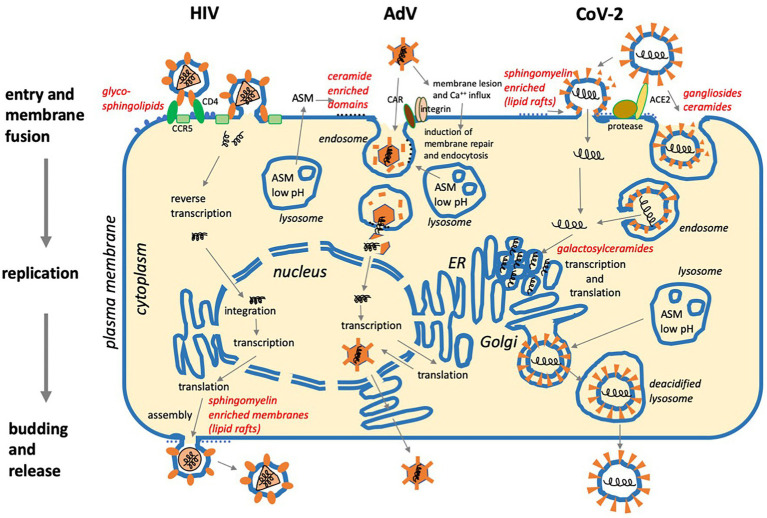
Three examples of viral replication cycles and involvement of sphingolipids: (1) Human immunodeficiency virus (HIV) binds to glycoshingolipids, CD4, and co-receptors CCR5 or CXCR4 prior to membrane fusion at the plasma membrane. After reverse transcription of the positive-strand RNA genome and integration in the cellular DNA, the virus exploits the cellular machinery for transcription and translation. Assembly and budding take place at the plasma membrane with the involvement of sphingomyelin-enriched membrane domains (lipid rafts). (2) Adenovirus (AdV) binds to CAR and integrins, then causes membrane lesions which induce Ca^++^ influx and membrane repair. This occurs with the help of lysosomes requiring ASM activity, which supports viral uptake by endocytosis. After the disintegration of the viral capsid, the viral DNA is transported to the nucleus, and after transcription and translation, viral particles are assembled in the nucleus and released. (3) SARS Coronavirus-2 (CoV-2) requires protease activity and binds to ACE2, which is supported by sialic acid in ganglioside-enriched membrane domains prior to cell entry *via* fusion at the plasma membrane or endocytosis and subsequent membrane fusion. Transcription and translation take place on the cytoplasmic face of the ER. Galactosylceramide supports viral replication. After assembly, virus particles traffic to lysosomes for egress *via* lysosomal exocytosis (For references, see main text).

It is known for several years now that membrane domains enriched in sphingolipids and cholesterol, also referred to as lipid rafts, are involved in cellular signal initiation as well as the direct structural support of viral entry by accumulating specific receptors. Besides the primary receptor CD4, glycosphingolipids (GSLs) present in these membrane microdomains, amongst them globo-triasylceramide (Gb3) and galactosylceramide (Gal-Cer), were found to interact with the human immunodeficiency virus (HIV) envelope proteins gp120 and gp41 to facilitate their interactions with chemokine receptors and to support its entry into CD4-negative cells as, for example, mucosal epithelial cells (Figure 2; Cook et al., 1994; Hammache et al., 1998a,b; Alfsen and Bomsel, 2002; Magerus-Chatinet et al., 2007; Yu et al., 2008; Dorosko and Connor, 2010; Lingwood et al., 2010). The importance of GSLs in HIV entry is further supported by its sensitivity to compounds affecting GSL biosynthesis, such as D-threo-1-phenyl-2-decanoylamino-3-morpholino-1-propanol (PDMP), which inhibits glucosyl-transferase activity (Puri et al., 2004), and to variations in cellular GSL content (Rawat et al., 2004). Interestingly, Gb3, when accumulating to high levels (for instance, in PBMCs of Fabry disease patients or certain cell lines), can also act as a resistance factor for human immunodeficiency virus type 1 (HIV-1) infection (Lund et al., 2005, 2009; Ramkumar et al., 2009; Harrison et al., 2010).

Glucosylceramide levels proved to be particularly important in regulating the uptake of viruses that rely on the late endosomal compartment to initiate membrane fusion and entry into the cytoplasm. These include influenza, Ebola, and vesicular stomatitis virus, the entry of which was sensitive to depletion of both anabolic and catabolic enzymes producing and processing glucosylceramide (Drews et al., 2019, 2020). Interaction with Gb4Cer (globotetraosylceramide, globoside) triggers viral capsid rearrangements of (the non-enveloped) parvovirus B19 required for subsequent steps of internalization into cells also expressing the erythropoietin receptor (Bonsch et al., 2010). Gangliosides (glycosphingolipids with one or more sialic acid residues linked to the sugar moiety) such as GD1a and GT1b or GM1 also serve as essential components for the entry of murine polyomavirus and SV40 into cells (Burckhardt and Greber, 2009; Luo et al., 2016). Furthermore, simultaneous engagement of gangliosides and 4-integrin was shown to promote endocytosis and microtubular trafficking of polyoma viral capsids by initiating PI3K, FAK/Src, and MAPK signaling pathways (O’Hara and Garcea, 2016). Obviously, GSLs can be of crucial importance in the uptake of certain viruses, which implies that interference with GSL biosynthesis might represent an interesting therapeutic option (Aerts et al., 2019).

### Ceramide Enriched Membrane Microdomains in Viral Uptake and Trafficking

Ceramide-enriched membrane domains condense into larger platforms in response to sphingomyelinase activation or ceramidase inhibition and are also sites of endocytic uptake of pathogens due to concentration of pathogen receptors or the action of membrane-proximal signaling complexes (Zha et al., 1998; Holopainen et al., 2000; Gulbins and Grassme, 2002; Gulbins et al., 2004; Bollinger et al., 2005; Grassme et al., 2007). Therefore, conditions favoring the generation of these domains create an environment enhancing viral infections. It has, for instance, been shown that the ability of CD300lf to support murine Norovirus entry depends on sphingolipid biosynthesis and ceramide generation. Furthermore, exogenous addition of ceramide restored susceptibility of serine palmitoyl-transferase deficient cells, which cannot synthesize ceramide themselves. This relied on both formation of ceramide enriched membrane domains and ceramide-induced conformational changes of surface resident CD300lf proteins (Orchard et al., 2018). Sphingomyelinase activation also supported pH and clathrin-dependent entry and replication of Japanese encephalitis virus (JEV) in tissue culture (Tani et al., 2010). Strikingly, sphingomyelin-synthetase-1 (SMS-1) generated sphingomyelin proved to be important in JEV attachment and subsequent infection, and SMS-1 deficiency in mice led to attenuation of the JEV infection *in vivo* (Taniguchi et al., 2016). Interestingly, rhinovirus raft interaction promoted biphasic activation of p38 MAPK in a RhoA-dependent manner, with late activation relying on viral replication (Dumitru et al., 2006).

In addition to sphingomyelin, acid sphingomyelinase (ASM) activity was also implicated in the early steps of Ebola virus infection. While sphingomyelin was required for attachment, viral particles strongly associated with surface displayed ASM indicating that viral interaction may occur in sphingomyelin-enriched membrane domains followed by ASM activation (Miller et al., 2012b). As for rhinovirus, receptors involved in ASM activation were not identified, and it also remained unclear whether ASM activation would be important in Ebola virus endocytosis and thereby rendering the endo/lysosomal cholesterol transporter Niemann-Pick C protein 1 (NPC1) accessible to the viral particle. NPC1 was identified as crucial for Ebola virus uptake by enabling fusion between viral and endosomal membranes (Carette et al., 2011; Cote et al., 2011). Thus, NPC1 acts as a receptor for the proteolytically activated viral envelope protein in an intracellular compartment rather than at the plasma membrane (Miller et al., 2012a).

Interaction with its surface receptors CAR and αvβ3 integrin causes limited uncoating of the non-enveloped adenovirus particle at the cell surface, which leads to exposure of the adenoviral protein-IV causing membrane lesions followed by Ca^2+^-influx promoting a wound repair process with subsequent lysosomal exocytosis (Figure 2). Along with that, ASM is activated and displayed at the surface and generates ceramide enriched membrane domains (Luisoni et al., 2015). These act to enhance viral endocytosis and recruit as well as to concentrate lytic protein-VI in endosomes, thereby catalyzing endosomal leakiness and finally rupture as required to release the viral capsid into the cytosol. Thus, adenovirus uses a positive feedback loop between virus uncoating and lipid signaling for efficient membrane penetration.

Acid sphingomyelinase (and neutral sphingomyelinase, NSM) activation was also observed after the interaction of dendritic cells (DC) with MV (Avota et al., 2011). In this case, the interaction of the viral glycoproteins with DC-SIGN on the cell surface induced the sphingomyelinase activation and subsequent ceramide release. Interestingly, this was also promoted by DC-SIGN ligation with specific antibodies or the ligand mannan, revealing that this reflected DC-SIGN signaling *per se* and was not MV-specific. Along with ASM, the MV entry receptor CD150 translocated from an intracellular storage compartment to the cell surface and thereby was made available to promote viral infection of dendritic cells. Whether or not CD150 translocation on the surface of dendritic cells may be important for pathogens other than MV has not been investigated. In murine macrophages, CD150 can also serve as a microbial sensor routing Gram-negative bacteria into phagocytic compartments (Berger et al., 2010).

The recent severe acute respiratory syndrome coronavirus type 2 (SARS-CoV-2) pandemic has initiated an intensive search for therapeutic approaches, including studies on drug repurposing. It was found that sphingolipid and cholesterol-enriched membrane microdomains (lipid rafts) are also associated with the uptake of SARS-CoV-2 involving the receptor ACE-2 (Figure 2; angiotensin-converting enzyme-2; Hoffmann et al., 2020; Wan et al., 2020; Yan et al., 2020). The spike protein of SARS-CoV-2 has been found to interact with the ganglioside GM1, which may mediate attachment to lipid rafts and facilitate the contact of the virus with its receptor ACE-2 (Fantini et al., 2020). By catalyzing the hydrolysis of sphingomyelin to ceramide, sphingomyelinases convert rafts into ceramide-enriched platforms (Bieberich, 2018). In this context, targeting the ASM activity by well-known inhibitors (Kornhuber et al., 2011) led to promising results. Both, genetic ASM ablation, as well as pharmacological ASM inhibition by fluoxetine (and other functional ASM inhibitors) reduced SARS-CoV-2 infection in several cell lines and primary nasal epithelial cells by preventing the formation of ceramide-enriched membrane platforms required for viral uptake (Carpinteiro et al., 2020, 2021; Zimniak et al., 2021). The accumulation of cholesterol and pH buffering downstream of the ASM inhibition in late endosomal compartments prevented the fusion of viral and late endosomal membranes required for viral uncoating (Schloer et al., 2020). For a recent review, see also Tornquist et al. (2021).

Ceramide generation, as observed after ASM activation, may also act antivirally at the level of uptake: Entry of HIV-1 into T cells, monocytes or macrophages was highly sensitive to compounds elevating levels of ceramides such as exogenous addition of long-chain ceramide (C_16_), because this prevents lateral diffusion of CD4 toward the chemokine co-receptors, and thus separates receptors and co-receptors (Finnegan et al., 2004, 2007; Rawat et al., 2008). In contrast, optimal HIV-1 gp41-mediated membrane fusion was found to be dependent on sphingomyelin synthase-2 activity, indicating that sphingomyelin rather than ceramide accumulation was important in this process (Hayashi et al., 2014). Similarly, the overall elevation of ceramides by bacterial sphingomyelinase interfered with the uptake of the hepatitis C virus (HCV) at the level of receptor segregation. CD81, a major entry factor, was partially internalized, and this and other components required for HCV entry, scavenger receptor B1 and claudin-1, were excluded from detergent-resistant microdomains (Voisset et al., 2008). Depletion of sphingomyelins and generation of ceramides also affects the entry of pseudorabies virus (Pastenkos et al., 2019), rubella virus (Otsuki et al., 2018), and influenza virus (Audi et al., 2020).

A further consequence of ASM activation is interference with actin dynamics in viral target cells. This affects, for example, drifting and surfing of receptors engaged by viruses along filopodia or on the cell surface (Ewers et al., 2005; Lehmann et al., 2005; Schelhaas et al., 2008), receptor clustering, and formation of and viral transmission by defined structures such as virological synapses or filopodial bridges (Sherer et al., 2007; Mothes et al., 2010). Moreover, actin-mediated membrane ruffling and blebbing are essential for macro-pinocytic uptake of vaccinia, picorna and adenoviruses (Mercer and Helenius, 2008, 2009). Thus, interference with actin dynamics would be expected to have a significant impact on viral uptake. In this context, it is noteworthy that the breakdown of actin cytoskeletal protrusions after ASM activation and subsequent ceramide accumulation were observed in MCF-7 breast cancer cells (Zeidan et al., 2008), and, NSM- and ASM-dependently, upon measles virus (MV) interaction with T cells (Gassert et al., 2009; Mueller et al., 2014).

## Intracellular Sphingolipid Interactions During Viral Replication

Individual sphingolipid species accumulating inside host cells may be favorable for either the host cell or the virus. Evidence for a protective role of ceramides was provided, for instance, in human lung epithelial cells where replicating, but not UV-inactivated, influenza A virus induced *de novo* biosynthesis of ceramide, which limited viral replication (Soudani et al., 2019) as previously also suggested for hepatitis and B virus (Tatematsu et al., 2011; Perera et al., 2012b). In contrast, sphingolipids including ceramides may also act pro-virally by supporting viral replication as revealed for HCV, West Nile virus (WNV), and Dengue virus (Perera et al., 2012a; Zhang et al., 2019). These are positive-strand RNA viruses known to extensively remodel cellular membranes into distinct compartments referred to as viral replication compartments (VRCs). VRCs act as platforms to concentrate viral proteins, assemble replication complexes, and protect those from recognition by innate immune defense mechanisms. This has been extensively studied with respect to sterols and glycerophospholipids (reviewed in Strating and van Kuppeveld, 2017), while the role of sphingolipids in this process is less well understood. Pharmacological inhibition of sphingomyelin biosynthesis interfered with replication of HCV and WNV, and sphingomyelin, glycosphingolipids, or ceramide, respectively, were detected in association with VRCs (Weng et al., 2010; Khan et al., 2014; Martin-Acebes et al., 2016).

If infected by an intracellular bacterial genus called Wolbachia, mosquitos are much less efficient in transmitting Dengue Virus to humans. Supporting a pro-viral role of sphingolipids in Dengue virus replication, all sphingolipid classes found to be enriched in Dengue-infected mosquito cells were depleted in the presence of Wolbachia, which obviously created an unfavorable lipid environment for the virus (Molloy et al., 2016). A comprehensive lipidomic study recently revealed significantly remodeled lipid composition in Huh7 cells upon infection with Zika virus (another flavivirus), which particularly affected sphingolipid subclasses (Leier et al., 2020). Inhibition of the sphingolipid biosynthesis interfered with viral replication in various cell types, while the exogenous supply of ceramide sensitized target cells for viral infection. Interestingly, ceramide was found to redistribute to Zika virus replication sites and interact with the viral non-structural protein 4B (NS4B), suggesting that ceramide flux takes part in VRC formation and activity.

Interestingly, a recent study identified sphingosine accumulation as a result of ceramide breakdown by acid ceramidase as a cell-intrinsic antiviral defense mechanism in macrophages. In these cells, the herpes simplex virus (HSV-1) was found to get trapped in endosomal compartments enriched for sphingosine and ablation of acid ceramidase promoted HSV-1 capsid export into the cytosol (Lang et al., 2020). Acid ceramidase expression was induced downstream of IRF-8 signaling, and in this model, sphingosine production proved to be the crucial effector for the protection of macrophages from infection *in vitro* and *in vivo*. Furthermore, sphingosine kinases and sphingosine-1-phosphate (S1P)-dependent signaling pathways support the replication of HSV-1 in endothelial cells (Graber et al., 2020).

**Figure 3:**
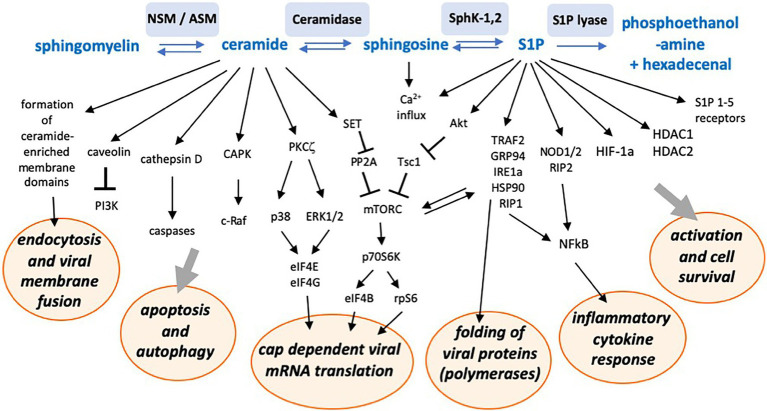
Schematic representation of a number of sphingolipid-associated signaling pathways and interactions with cellular functions potentially supporting or inhibiting viral replication. NSM, neutral sphingomyelinase; ASM, acid sphingomyelinase; SphK-1,2, sphingosine kinases 1 and 2; S1P, sphingosine-1-phosphate; CAPK, ceramide activated protein kinase; PKC, protein kinase C; PP2A, protein phosphatase 2 A; TRAF2, tumor necrosis factor associated factor 2; GRP94, glucose-regulated protein 94; IRE1a, inositol requiring enzyme 1 alpha; Hsp90, heat shock protein 90; RIP1, receptor-interacting protein 1; HDAC, histone deacetylase; and HIF-1a, hypoxia inducing factor 1 alpha.

Sphingosine-1-phosphate metabolizing enzymes and associated sphingolipids may provide targets for antiviral strategies against a number of viruses (summarized in Wolf et al., 2019). Non-structural 3 (NS3) protein of bovine viral diarrhea virus (BVDV), a close relative of HCV, was found to bind to and inhibit sphingosine kinase 1 (SphK1) and to be important for efficient viral replication (Yamane et al., 2009). In contrast, activation of neutral ceramidase and SphK1, with resulting S1P generation and AKT and ERK activation (see also Figure 3), supported replication of the respiratory syncytial virus (RSV) in lung epithelial cells (Monick et al., 2004). Sphingosine kinase 2 (SphK2), which, unlike its more extensively studied isoform SphK1, additionally possesses a nuclear localization signal (NLS) and a nuclear export signal (NES), was observed to co-localize with the replication complex of Chikungunya virus and pharmacological inhibition of its kinase activity reduced viral infection (Reid et al., 2015).

A pro-viral role of sphingosine kinase/S1P was also seen for influenza A (IAV), measles (MV), and human cytomegalovirus replication (HCMV; Seo et al., 2010; Vijayan et al., 2014; Zilch et al., 2018). Thus, SphK1 overexpression enhanced IAV protein synthesis and synthesis of viral progeny (Seo et al., 2013), while SphK1 inhibition reduced viral replication by interfering with the nuclear export of viral RNPs (Vijayan and Hahm, 2014). IAV infection itself activates SphK1 (Seo et al., 2013) and thereby stimulates the NFκB pathway, promoting viral RNA synthesis (Kumar et al., 2008). Similar observations were made for MV infection, where SphK1 inhibition impaired viral protein expression and suppressed MV-induced activation of NFκB in certain cell lines (Vijayan et al., 2014). Also, in its natural target cells, primary human peripheral blood cells, inhibition of both acid ceramidase or sphingosine kinase impaired MV replication (Grafen et al., 2019). Rather than acting on a viral target directly, the latter particularly affects components of the cellular machinery, including Hsp90 and mTORC1, required for efficient MV replication (Bloyet et al., 2016; Tiwarekar et al., 2018).

A negative effect of elevated S1P concentrations induced by the SphK activator K6PC-5 has been observed for Ebolavirus infections. The effect was independent of S1P receptors and appears to be mediated intracellularly, affecting the viral entry process (Imre et al., 2021). Thus, sphingosine kinases and intracellular S1P can have differential effects on virus replication depending on the virus and the target cell. By activating NFκB *via* interaction with tumor necrosis factor associated factor (TRAF2; Alvarez et al., 2010) or NOD1/2 (Pei et al., 2021), S1P also induces an inflammatory cytokine response.

In addition to its intracellular effects, S1P also acts on S1P receptors influencing immune cell trafficking and other functions of the immune system. In this respect, it is interesting that studies performed in 3D cultures modeling the respiratory tract supported a key role of MV-induced S1P to promote fast ameboid migration of dendritic cells toward the lung epithelial cell layer. As dendritic cells function as cellular ferries of MV, this may be important for transmitting MV during viral exit from the infected individual (Derakhshani et al., 2019).

These findings show that predominantly ceramide and S1P are important signaling molecules regulating not only the cell metabolism, but which are also intrinsically tied to the capacity of a target cell to replicate viruses. To provide an overview over the multiplicity of involved pathways, we schematically summarized sphingolipid-associated signaling pathways and intracellular consequences potentially affecting viral replication in Figure 3. The figure may give an impression of the complex network involved. It is likely that within the next years, more interactions with more viruses will be revealed.

## Sphingolipids in Viral Assembly and Budding

As indicated above for VRCs, the biogenesis of lipid structures can be intimately coupled to viral replication and/or assembly. The latter process has been intensely studied for HCV and Dengue virus, where biogenesis of lipid droplets was shown to play a crucial role in the initiation of viral assembly (Alvisi et al., 2011; Fischl and Bartenschlager, 2011). Interestingly, ceramide transfer protein (CERT) was required for HCV maturation, suggesting an important contribution of the sphingolipid pathway in flavivirus replication (Amako et al., 2011). SARS CoV-2, as a further positive-strand RNA virus, uses an unusual egress mechanism involving lysosomal exocytosis (Ghosh et al., 2020). Newly synthesized viral particles traffic from the ER and the ER-Golgi intermediate compartment to lysosomes, which induces deacidification and inactivation of lysosomal enzymes (Figure 2).

The membrane patch where viral assembly occurs defines the composition of the viral particle’s envelope membrane. Initially established as highly relevant in membrane model systems, the importance of lipid-based protein sorting in mammalian cell membranes has, in fact, been established by pioneering studies on HIV-1 biogenesis (Sengupta et al., 2019; Sengupta and Lippincott-Schwartz, 2020). Compared to the lipid composition of the cell membrane in general, the HIV-1 particle substantially differs in its lipid composition, i.e., it is selectively enriched for sphingomyelin and dihydro-sphingomyelin, while ceramides are barely represented (Figure 2; Brugger et al., 2006; Lorizate et al., 2013). This suggested that the viral core either selects already existing lipid species or actively remodels lipid composition of host cell membranes during the assembly and subsequent budding process. It was only after techniques had become available to visualize and trace single virus assembly by quantitative live-cell imaging that, driven by oligomerized HIV-1 Gag protein at the inner membrane leaflet, formation of ordered membrane domains orchestrating lipid and protein composition could be demonstrated (Favard et al., 2019; Sengupta et al., 2019). The molecular mechanisms induced by the viral core described in these and earlier elegant studies include modulation of membrane curvature, partitioning of lipid phases and, sequentially, sorting of proteins. Moreover, these studies highlight the importance of trans-bilayer coupling of lipid composition through acyl chain interactions, through which phase separation of the outer membrane leaflet assembly site is achieved (Carlson et al., 2008; Briggs et al., 2009).

Finally, the lipid composition of the enveloped viral particle may substantially affect its infectivity. Treatment of bovine herpesvirus particles with bacterial sphingomyelinase, but not that of the target cells, reduced viral entry suggesting that the sphingomyelin content of the particle is important. However, this cannot be generalized because pseudorabies virus entry was sensitive to sphingomyelinase at the level of the host cell and not the particle, while HSV-1 entry was insensitive to sphingomyelinase exposure of either the cell or viral membrane (Pastenkos et al., 2019). Again, in contrast, sphingomyelin was found to be important in influenza virus infection both at the level of the viral particle and the host cell (Audi et al., 2020). As revealed for HIV-1 and Ebola virus particles, viral uptake into dendritic cells is substantially enhanced upon recognition of sialylated gangliosides anchored to viral membranes by Siglec-1 on the dendritic cells (Puryear et al., 2013; Perez-Zsolt et al., 2019).

The infectivity of viral particles budding into intracellular compartments may also be determined by their sphingolipid composition. Thus, the HCV RNA-dependent polymerase NS5B and p7 protein cooperatively promote infectivity of the viral particle by decreasing its sphingomyelin content (Aligeti et al., 2015). The morphogenesis of BVDV, which is budding into the ER, also involves a lipid sorting mechanism (Callens et al., 2016). BVDV particles were found to be particularly enriched for cholesterol, sphingomyelin, and hexosyl-ceramide, with both cholesterol and sphingomyelin being of functional importance for attachment and entry of the virus.

## Conclusion

Many chemical compounds affecting sphingolipid metabolism have been tested in animal models *in vivo* or are already in clinical use against diseases as divers as cancer and multiple sclerosis. For example, the S1P analog FTY720 (Wolf et al., 2019; Wang et al., 2020) is approved for the treatment of multiple sclerosis, and inhibitors of ASM and NSM are in use against a number of diseases including Parkinson’s disease and forms of depression (Kornhuber et al., 2014). Other inhibitors targeting ceramidase or SphK1 and -2 are in use or under investigation against certain tumors (Don et al., 2014). As such inhibitors have not only structural consequences but also affect signaling and the cellular metabolism, it is not surprising that also the replication of viruses is affected. So far, the effectivity of such inhibitors against viral infections has mostly been investigated in cell culture experiments. Yet, the knowledge concerning potential antiviral applications and side effects *in vivo* (especially in humans) is still very limited. Side effects can only be studied in a whole organism in the presence of a functional immune system. Often, effects on the immune system dominate the overall antiviral response, as for example, in the case of persistent lymphocytic choriomeningitis virus (LCMV) infection of mice, where inhibition of the SphK2 stimulates the T cell response and elimination of the infection (Studstill et al., 2020). Especially with respect to SARS-CoV-2 infection, it will be interesting to see if repurposed drugs affecting sphingolipid metabolism will be of use to reduce viral replication *in vivo* and accelerate viral clearance.

## Author Contributions

All authors listed have made a substantial, direct and intellectual contribution to the work, and approved it for publication.

## Funding

We thank the German Research Foundation (DFG) for funding grants SCHN 320/24-2, BE 4080/3-2, and GRK2581/1(P3), the Vogel Stiftung Dr. Eckernkamp, and the funding program “Open Access Publishing of the University of Würzburg.”

## Conflict of Interest

The authors declare that the research was conducted in the absence of any commercial or financial relationships that could be construed as a potential conflict of interest.

## Publisher’s Note

All claims expressed in this article are solely those of the authors and do not necessarily represent those of their affiliated organizations, or those of the publisher, the editors and the reviewers. Any product that may be evaluated in this article, or claim that may be made by its manufacturer, is not guaranteed or endorsed by the publisher.
